# Rethinking Nature’s Pharmacy: AI Era and Natural Product Drug Discovery

**DOI:** 10.3390/ph19020301

**Published:** 2026-02-11

**Authors:** Yipaerguli Paerhati, Alifeiye Aikebaier, Dilihuma Dilimulati, Alhar Baishan, Nazhakaiti Yusufujiang, Xiaoxiao Qiu, Yilixiati Wusiman, Wenting Zhou

**Affiliations:** 1 Department of Pharmacology, School of Pharmacy, Xinjiang Medical University, Urumqi 830017, China; yipaerguli@stu.xjmu.edu.cn (Y.P.); alifeiye@stu.xjmu.edu.cn (A.A.); dilihuma@stu.xjmu.edu.cn (D.D.); alhar@stu.xjmu.edu.cn (A.B.); nzkt0110@126.com (N.Y.); x485624574@163.com (X.Q.); xia6262025@163.com (Y.W.); 2 Xinjiang Key Laboratory of Natural Medicines Active Components and Drug Release Technology, Urumqi 830017, China; 3 Xinjiang Key Laboratory of Biopharmaceuticals and Medical Devices, Urumqi 830017, China; 4 Engineering Research Center of Xinjiang and Central Asian Medicine Resources, Ministry of Education, Urumqi 830017, China

**Keywords:** artificial intelligence, natural products, drug discovery, machine learning, virtual screening, de novo design

## Abstract

Natural products (NPs) have long been a cornerstone of pharmaceutical innovation, contributing to approximately 50% of FDA-approved drugs over the past four decades. However, traditional NP drug discovery faces significant hurdles, including laborious isolation processes, biodiversity constraints, and low hit rates in high-throughput screening. These hurdles often extend the development timelines to 10–15 years with costs exceeding $2 billion per drug. Artificial intelligence (AI) emerges as a transformative force, leveraging machine learning (ML), deep learning (DL), and generative models (Gen. AI) to expedite these processes. AI facilitates virtual screening of vast chemical libraries, predicts molecular interactions with unprecedented accuracy, and designs novel NP-inspired scaffolds, potentially reducing discovery time by up to 70%. This interdisciplinary approach not only addresses unmet medical needs but also aligns with global sustainability goals, potentially increasing success rates from <1% in traditional pipelines to over 10%. Ultimately, AI hints at revitalizing NP drug discovery, fostering innovative, eco-friendly therapeutics. This study reviews recent advancements in AI applications for NP drug discovery, including the challenges such as NPs representing only ~5% of screened compounds in many datasets, interpretability issues in “black-box” models, and ethical concerns over bioprospecting in biodiverse regions.

## 1. Introduction

### 1.1. Natural Products (NPs): A Historical Foundation of Medicine

Natural products (NPs), also referred to as bioactive small molecules originating from sources such as plants, animals, fungi, and microorganisms, have constituted the cornerstone of medical practice for thousands of years [1]. Originating from traditional practices such as Ayurveda, Traditional Chinese Medicine, and ethnopharmacological traditions of indigenous communities, these biologically derived compounds have consistently yielded structurally diverse molecules with significant therapeutic potential [2]. The earliest recorded application of natural therapeutics can be traced to ancient Mesopotamia, where cuneiform tablets dated to approximately 2600 B.C. document the medicinal use of cypress and myrrh oil for the treatment of various disorders [3]. This longstanding empirical knowledge has been continuously integrated into modern pharmacology. Prior to the advent of combinatorial chemistry and high-throughput screening platforms, over 80% of clinically used drugs were either directly derived from NPs or structurally inspired by them [4]. Comprehensive studies reveal that from 1981 to 2019, approximately one-third of small-molecule drugs approved by the U.S. Food and Drug Administration were either natural products or their direct derivatives. This proportion increases to nearly 50% when including synthetic compounds designed based on natural product frameworks [1]. This historical dependence highlights the unparalleled potential embedded in nature’s chemical diversity, refined through an evolutionary process over time to interact selectively and effectively with human biological systems [5].

Several transformative discoveries prove the foundational role of NPs in shaping contemporary medicine. Morphine, an opioid alkaloid isolated from the opium poppy (Papaver somniferum) in 1803, is an illustrative and prototypical example. Morphine (scaffold of morphine) has enabled the development of more than 70 structurally related therapeutics that include codeine, widely employed as an antitussive agent, and naltrexone, a key intervention for opioid dependence [6]. Likewise, the serendipitous discovery of penicillin by Alexander Fleming in 1928, originating from the Penicillium fungus, heralded the antibiotic era and irreversibly transformed medical practice [7]. Penicillin itself and more importantly its derivatives rapidly became indispensable for the treatment of previously fatal bacterial infections, potentially reducing mortality from pneumonia and other respiratory illnesses while facilitating the safety of surgical interventions [7].

The contribution of NP-derived therapeutics further extends to oncology and infectious diseases. Paclitaxel (Taxol), a diterpenoid isolated in the 1960s from the bark of the Pacific yew tree, emerged as a paradigm-shifting anticancer agent [8]. By stabilizing microtubules and thereby blocking mitotic progression, paclitaxel provided an innovative therapeutic option for ovarian cancer, particularly in drug-resistant cases [9]. Similarly, the discovery of artemisinin from Artemisia annua in 1972 revolutionized malaria treatment [10]. Artemisinin’s potent antimalarial activity prompted the World Health Organization to endorse artemisinin-based combination therapies (ACTs) as the global standard of care against drug-resistant malaria. Collectively, these landmark advances underscore the intrinsic strengths of NPs, namely their remarkable structural diversity, pronounced biological activity, and novel modes of action, which enable modulation of multiple molecular pathways and provide unique scaffolds for drug discovery that are often absent from synthetic compound libraries [11].

Overall, new molecular entities (NMEs), structurally unique pharmaceutical compounds that are significantly different from their existing counterparts, have an exceedingly low approval rate by the U.S. Food and Drug Administration (FDA), with only approximately 0.01% of candidates achieving market authorization [12].

### 1.2. Bottlenecks of Traditional NP Drug Discovery

Despite the long-standing success of natural products (NPs) in drug discovery, the pharmaceutical sector experienced a pronounced decline in NP-driven research beginning in the 1990s [13]. This transition was not attributable to a lack of therapeutic promise but rather to a set of inherent technical and logistical challenges that rendered the conventional discovery process inefficient, costly, and often unpredictable. These obstacles diminished the competitiveness of NP research relative to the emerging high-throughput screening and combinatorial chemistry platforms, which at the time offered the prospect of rapid and scalable drug discovery [13,14].

**Figure 1:**
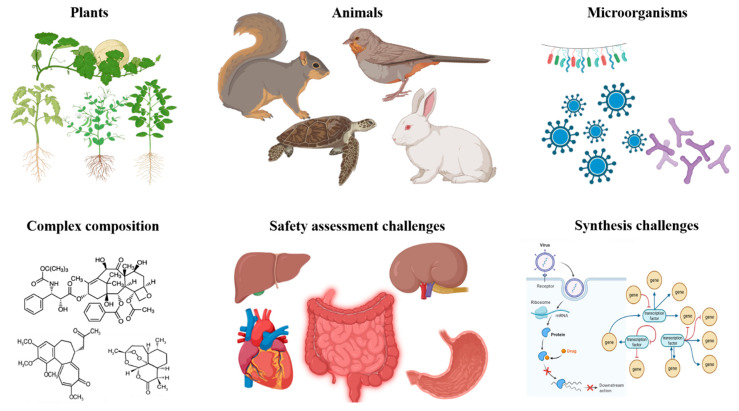
Challenges in the development of natural product-derived agents: from source complexity to safety and synthetic hurdles.

Among the most formidable hurdles is the technical complexity associated with NP isolation and characterization [1]. Natural extracts, particularly from plants, represent chemically intricate matrices comprising diverse primary and secondary metabolites, which makes the purification of an individual bioactive compound a labor-intensive, time-demanding, and resource-intensive endeavor [14]. Moreover, the sophisticated architectures of many NPs, frequently comprising cyclic or semi-rigid scaffolds enriched with multiple stereogenic centers, pose significant challenges for structural elucidation as well as for reproducible large-scale synthesis [15]. Such structural intricacy often correlates with suboptimal physicochemical properties; many otherwise promising NPs exhibit poor solubility, chemical instability, and inadequate bioavailability, all of which present critical barriers to successful clinical translation [16]. These findings are illustrated in Figure 1.

Another recurrent limitation in NP research is dereplication, defined as the inadvertent re-isolation of compounds already described in the scientific literature. This redundancy not only consumes substantial resources but also delays the identification of genuinely novel chemical entities [17]. Furthermore, while NPs represent a rich reservoir of pharmacologically active molecules, they may also manifest intrinsic toxicities. For instance, hepatotoxicity has been documented for comfrey, whereas Hypericum perforatum (St. John’s Wort) is associated with clinically significant drug–drug interactions [18]. Such risks necessitate rigorous toxicological evaluation, thereby introducing additional complexity, time, and cost into the development pipeline. Finally, the sustainability of NP-based drug discovery remains a pressing concern. The restricted availability of certain rare or endangered species, coupled with the typically low yields of bioactive constituents, presents profound ethical and ecological challenges, impeding the scalability and feasibility of clinical production [1].

### 1.3. AI: The Catalyst for a New Paradigm

The decline of natural product (NP)-oriented research in the late 20th century reflected a rational response to the prevailing economic and technological constraints of the pre-artificial intelligence (AI) era. However, the emergence of AI has fundamentally altered this landscape, acting as a powerful catalyst for the resurgence of interest in nature’s chemical space [19,20]. The analytical and predictive capabilities of AI, particularly through machine learning (ML) and deep learning (DL) frameworks, provide the computational infrastructure required to address the very barriers that previously hindered NP research [19]. By enabling rapid data processing, robust predictive modeling, and even the de novo design of novel molecular architectures, AI substantially accelerates the discovery process [20,21]. The integration of AI into NP drug discovery thus represents more than an incremental methodological refinement; it constitutes a transformative operational and conceptual paradigm shift that restores both the economic feasibility and scientific attractiveness of NP exploration [19,20]. The following table (Table 1) consolidates this central argument by mapping the principal historical challenges of NP drug discovery to contemporary AI-driven strategies and methodologies, thereby illustrating the stimulating potential of AI in revitalizing this domain.

## 2. AI in Initial Discovery and Identification of NP Leads

### 2.1. Unearthing NPs Through Omics Mining and Textual Data

Artificial intelligence (AI) is reshaping the early stages of drug discovery by enabling systematic interrogation of the vast, largely unexplored chemical diversity encoded within natural sources. This transformation is driven primarily through two complementary strategies: omics-based mining and the computational analysis of traditional knowledge. The advantage of AI in this context extends well beyond acceleration; its core strength lies in the ability to integrate and interpret heterogeneous, multimodal datasets at scales unattainable by conventional human-driven analysis, thereby generating a more comprehensive and interconnected view of the natural product (NP) landscape. One of the most transformative applications has been the exploration of the so-called “microbial Pandora’s box” through multi-omics integration, encompassing genomics, proteomics, and metabolomics [25]. Within this domain, genome mining leverages AI to identify biosynthetic gene clusters (BGCs) embedded in genomic sequences, molecular blueprints that encode secondary metabolite biosynthesis. Advanced tools such as DeepBGC have significantly outperformed traditional rule-based methods, achieving predictive accuracies of approximately 80% compared with only 60% for earlier approaches [26]. The true power of AI emerges when these disparate omics layers are integrated. Computational platforms such as NPLinker and GNPS directly connect genomic BGCs with mass spectrometry (MS)-derived metabolomic profiles, effectively linking biosynthetic potential to empirically observed chemical outputs [27]. Such integrative analyses uncover previously inaccessible relationships between genetic capacity and metabolite production, representing a major leap beyond what was previously achievable.

Similarly, a team of researchers at Westlake University, China has developed an AI-driven web-based molecule sharing platform named ShennongAlpha for the intelligent management, acquisition, and translation of compounds extracted or reported from Natural Medicine [28]. The platform holds over 14,593 pieces of compound/phytochemical information as per the last update reported [29]. The platform includes Shennong Dialog and Shennong Nomenclature, which uses NMT-CPT (Neural Machine Translation with Coreferential Principal Term) to enable standardized translation between Chinese and English, eliminating language barriers, as well as generates standardized systematic names while automatically producing a graph to link the molecules with their association to diseases [29].

By systematically extracting association rules between canonical biosynthetic pathways and their corresponding chemical structures, using publicly available resources such as MIBiG and antiSMASH, computational frameworks like antiSMASH have become indispensable in natural product research. To date, thousands of putative biosynthetic gene clusters (BGCs) have been detected across microbial genomes and subsequently cataloged in public repositories. The functional interpretation and assessment of the novelty of these predicted BGCs necessitates comparative analysis with a reference set of experimentally validated clusters of known activity. To address this need, the Minimum Information about a Biosynthetic Gene cluster (MIBiG) standard and repository was launched in 2015 to provide a structured framework for the storage and curation of characterized BGCs [30]. The release of MIBiG 2.0 introduced significant improvements to its infrastructure, data content, and accessibility, incorporating 851 newly curated BGCs over the past five years. Furthermore, extensive expert-driven manual curation has substantially enhanced the accuracy of functional annotations, thereby enabling the development of comprehensive predictive pipelines that bridge gene sequences to their respective molecular products [31]. These advances underscore the capacity of machine learning approaches to integrate multi-omic datasets, including genomic, transcriptomic, and metabolomic information, for the identification and prioritization of novel drug targets.

In parallel, AI-driven natural language processing (NLP) and Large Language Models (LLMs) have emerged as a transformative tool for extracting knowledge embedded within vast repositories of unstructured text [32]. This encompasses diverse sources, such as ancient manuscripts, ethnobotanical records, and contemporary scientific literature [33]. By parsing and structuring this heterogeneous data, NLP systems can systematically catalog medicinal plant species, their historical therapeutic uses, and reported pharmacological effects [34,35]. What was once a painstaking manual process has now become scalable and data-driven. Critically, it is critically important to mention when this textual knowledge is cross-referenced with chemical and omics datasets. AI enables prioritization of high-value NP candidates for experimental validation [23]. This creates a powerful bidirectional feedback system by providing insights from traditional knowledge-directed AI-based exploration toward promising chemical space, while multi-omics validation provides mechanistic grounding by identifying and characterizing the bioactive compounds responsible for these effects.

Traditional Chinese Medicine (TCM), grounded in concepts such as “Qi” and the yin–yang equilibrium, has been practiced for centuries through modalities including acupuncture, herbal therapy, and dietary interventions [36,37]. The complexity of its multi-herb, multi-compound formulations has traditionally limited mechanistic understanding. Recent advances in artificial intelligence (AI) now enable systematic elucidation of bioactive constituents and therapeutic mechanisms using data mining, pattern recognition, and predictive modeling, thereby reframing TCM within the context of systems pharmacology and network medicine [38,39].

A key development in this field is the TCMBank database, established by CHEN Yuqian’s team, which integrates 9192 herbal medicines, 61,966 unique ingredients, 15,179 targets, and 32,529 diseases, transitioning TCM research from experience-based practice to data-driven discovery [40]. TCMBank addresses three major challenges: (i) a human–machine annotation system that improves curation efficiency 17-fold; (ii) multi-source heterogeneous data fusion via deep transfer learning to unify terminologies from classical texts; and (iii) AI-assisted models that reveal mechanisms of complex formulas and support applications such as drug–target prediction, lead compound design, safety evaluation of Chinese–Western medicine combinations, retrosynthetic analysis, and vaccine development [41,42].

The application of AI to many African, Amazonian, or Indigenous traditions highlights the fundamental challenges of data scarcity, shifting its primary role from high-level prediction to intelligent data curation and preservation [43]. Tools are being developed to systematically document plant use (e.g., the UmzimbaOmhle app for South African plants), with the aim of constructing structured, machine-readable datasets that can be used for future predictive discovery [44].

Discovery in the modern frontier of marine and microbial metabolites is fueled by dedicated omics databases; for example, the microbial database SBC and the marine antimicrobial database AntiMarin. AI models trained on these resources can be used to predict novel bioactive structures from genomic or metabolomic data, effectively mining the chemical innovations of entire ecosystems [45].

By integrating AI with TCM and linking traditional knowledge to multi-omics and systems pharmacology, this paradigm establishes a powerful translational framework for precision medicine and accelerates drug discovery.

### 2.2. Accelerating Characterization and Dereplication of NPs

Following the acquisition of a natural extract, artificial intelligence (AI) has markedly accelerated the traditionally laborious process of structural elucidation. Contemporary AI and machine learning (ML) algorithms are now seamlessly integrated with advanced analytical platforms, including nuclear magnetic resonance (NMR), high-performance liquid chromatography–mass spectrometry (HPLC–MS), and gas chromatography–mass spectrometry (GC–MS), thereby enhancing the speed, precision, and interpretability of experimental data [19,46,47,48]. This synergistic integration has transformed structural analysis into a significantly more efficient workflow, enabling the practical implementation of high-throughput screening pipelines for natural products (NPs) [19]. Notable progress has been demonstrated through the development of deep learning-based frameworks. For instance, DN-Unet, a deep neural network, has been shown to potentially improve the signal-to-noise ratio of NMR spectra (by over 200-fold), recovering weak spectral peaks that are otherwise masked by noise [49]. In parallel, the DP4-AI and DP5-AI platform automates the analysis and assignment of raw NMR data, achieving processing speeds up to 60 times faster than conventional manual approaches while significantly reducing dependence on expert interpretation [50]. These advances enable the rapid structural identification of compounds within complex mixtures, thereby minimizing the necessity for extensive and resource-intensive purification procedures. AI-driven virtual screening (AI-VS) strategies are generally classified into ligand-based virtual screening (LBVS) and structure-based virtual screening (SBVS). LBVS leverages structure–activity relationships to predict new bioactive compounds using graph neural networks for three-dimensional molecular feature extraction (AUC > 0.90), geometric deep learning to optimize pharmacophore models, and Transformer-based architectures for ADMET prediction. SBVS, in turn, utilizes three-dimensional target structures for precise molecular interaction modeling, with advanced docking algorithms such as DiffDock addressing limitations in conformational sampling [51,52].

To facilitate natural product research, the HERB database (Ben Cao Zu Jian) was established as a large-scale TCM resource linking Chinese herbal medicines with modern pharmacology. Through the reanalysis of 6164 gene expression profiles from 1037 experiments and integration with CMap, HERB mapped TCM ingredients to 2837 modern drugs. Additionally, curated datasets linked 12,933 targets and 28,212 diseases to 7263 medicinal materials and 49,258 compounds across six types of pairwise relationships [53,54,55]. While HERB provides a powerful platform for TCM modernization and rational drug development, limitations remain in data coverage and novel toxicity prediction, highlighting the need for multi-omics integration and causal inference frameworks [54].

One of the most persistent bottlenecks in NP discovery is dereplication, the repeated re-identification of previously known molecules, which results in considerable inefficiency and redundancy [56]. AI directly addresses this limitation through sophisticated pattern recognition, classification, and clustering methodologies that can rapidly compare newly generated spectral profiles against curated reference databases [56,57]. For example, unsupervised learning algorithms such as K-means clustering can organize structurally related compounds, facilitating a clearer assessment of chemical diversity within libraries and prioritizing molecules with genuine novelty for downstream investigation [58]. Complementary tools, such as NaturePred, employ natural language processing (NLP)-based approaches to predict NP classes with high accuracy, further optimizing dereplication and candidate prioritization [59].

These breakthroughs in molecular semantic vectorization, 3D structure–activity modeling, and accurate free energy prediction have systematically increased success rates in active compound identification. By advancing beyond conventional library-matching techniques, AI establishes a more intelligent, scalable, and resource-efficient strategy for identifying structurally novel and pharmacologically promising natural product leads [60].

### 2.3. Translational Pathways and Regulatory Considerations for AI in NP Discovery

The translation of AI-driven NP discovery from academic research to a component of the regulated drug development process necessitates rigorous engagement with regulatory science, robust validation, and honest assessment of translational readiness. Regulatory bodies like the FDA and EMA currently provide guiding principles rather than prescriptive rules for AI usage in discovery, emphasizing transparency, scientific rigor, and robustness [61]. For instance, the FDA’s discussion paper on AI/ML usage in drug development highlights the importance of creating a “predetermined change control plan” for models that learn and adapt, which is directly relevant to active learning pipelines in NP optimization [62]. This aligns with the lifecycle approach to model validation advocated in guidelines like ICH Q9, which moves beyond one-time testing to ongoing performance monitoring and management [63].

Consequently, validation strategies must evolve. Beyond reporting cross-validation accuracy, models intended for decision support must undergo prospective validation by using a locked version on external, blinded datasets that simulate real-world use. Furthermore, documenting the model’s “applicability domain”—the chemical and biological space within which its predictions are reliable—is crucial to prevent spurious extrapolation to novel NP scaffolds outside the distribution of the training data. A practical framework for assessing maturity is the Technology Readiness Level (TRL). While most AI-for-NP tools reside at a TRL of 3–4 (experimental proof-of-concept), achieving a TRL 6–7 (prototype validation in a relevant industrial environment) requires demonstrating interoperability with existing lab informatics systems, reproducibility across batches, and a tangible impact on key metrics [64].

**Figure 2:**
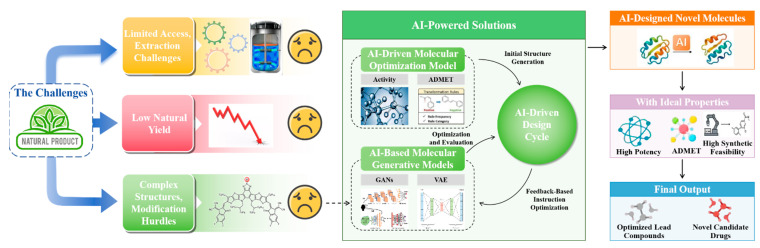
AI-powered strategies for addressing natural product drug development challenges and enabling rational molecule design.

The journey from a published algorithm to an adopted tool is bridged by addressing these translational gaps. This includes developing standardized formats for NP-omics data, benchmarking challenges under controlled conditions, and fostering pre-competitive collaborations to validate tools on proprietary industry datasets. Ultimately, rather than constraints, these frameworks should be seen as catalysts for building trustworthy, impactful, and scalable AI solutions that can reliably contribute new natural product-based therapies [28]. Further details are provided in Figure 2.

## 3. Preclinical Development: From Target Engagement to Lead Optimization

### 3.1. Predicting Targets and Mechanisms of Action

In the preclinical phase, artificial intelligence (AI) is redefining drug discovery by transforming it from a predominantly empirical, trial-and-error process into a systematic, data-driven endeavor, wherein the interactions of candidate compounds with biological systems are computationally inferred. This stage, referred to as target deconvolution, presents particular challenges in the context of natural products (NPs), which frequently exhibit pleiotropic or multi-target activities as a consequence of their evolutionary adaptations [65]. AI methodologies are uniquely suited to address this complexity by integrating multi-omics datasets with network-based analytical frameworks to elucidate novel therapeutic targets and pathways [66,67]. This provides a more holistic characterization of a compound’s mechanism of action (MoA), extending beyond the identification of a single, discrete molecular target.

To facilitate this process, several AI-driven platforms have been developed. For instance, the SPiDER (self-organizing map-based prediction of drug equivalence relationships) algorithm can identify putative molecular targets by analyzing the physicochemical properties of a compound and mapping them against those of known drugs, even in cases lacking strong structural similarity [68]. Likewise, the STARFish (stacked ensemble target fishing) framework employs ensemble learning strategies to predict the interactions of small molecules with a broad spectrum of targets, with particular utility in NP target identification [69]. These computational approaches permit the generation of in silico hypotheses regarding a compound’s MoA, thereby streamlining the prioritization of candidates for subsequent experimental validation, which is often resource-intensive and time-consuming [70].

For instance, a multimodal machine learning framework was applied to identify anti-Alzheimer’s disease (AD) compounds within complex TCM formulations. Four deep neural network (DNN) models—trained at both the disease and target levels (acetylcholinesterase, monoamine oxidase-A, and 5-HT6 receptors)—successfully predicted candidate compounds, which were experimentally validated at the enzymatic, cellular, and animal levels. Molecules such as 2,4-di-tert-butylphenol and elemene exhibited strong inhibitory effects on AD targets, while compounds including α-asarone penetrated the blood–brain barrier and enhanced microglial β-amyloid clearance, confirming the therapeutic potential of AI-driven predictions [71,72,73,74,75,76,77]. Similarly, a team from the School of Pharmacy, Fudan University integrated geometry-aware deep learning with biological validation to screen over 300,000 natural products, identifying bifunctional compounds that simultaneously regulated lipid membranes and targeted Glut1. These were incorporated into a liposome-based delivery system, improving tumor targeting and therapeutic efficacy in preclinical models [78].

### 3.2. Assessing Bioactivity, ADMET, and Toxicity Profiles

One of the principal factors underlying the high cost and low success rates of conventional drug development is the substantial attrition of candidate molecules during both preclinical and clinical evaluations, largely attributable to unfavorable pharmacokinetic characteristics or toxicological liabilities [79]. Artificial intelligence (AI) is an efficient and cost-effective tool for addressing this issue, enabling the large-scale in silico screening of compound libraries for their Absorption, Distribution, Metabolism, Excretion, and Toxicity (ADMET) profiles; this substantially decreases the number of molecules that require synthesis and experimental testing, thereby streamlining and accelerating the overall drug development pipeline [80].

A representative example of this approach is ADMET-AI, a web-based platform that leverages the Chemprop-RDKit graph neural network architecture to predict 41 distinct ADMET parameters with high accuracy. This system allows thousands of candidate compounds to be evaluated rapidly, benchmarking their predicted pharmacokinetic and safety properties against those of approved drugs cataloged in resources such as DrugBank, and thus providing a contextual framework for assessing both safety and druggability [81,82,83].

Qi Yang et al. developed a machine learning model for hepatotoxicity prediction, validated with 56 chemical constituents of Gardenia jasminoides [84]. Their results revealed the dualistic nature of its hepatotoxic components, which exert therapeutic benefits at specific doses while inducing toxicity at others. This work highlights the pivotal role of artificial intelligence (AI) in ADMET prediction, as it enables focused experimentation, reduces clinical attrition rates, and lowers drug development costs [85].

To expand on this, Prof. Cao Dongsheng’s team has made substantial contributions to small-molecule drug-likeness prediction and Lead Optimization, advancing ADMET modeling and drug discovery paradigms. The ADMETlab platform is a notable example, which has developed from ADMETlab (2018) to ADMETlab 2.0 (2021), and most recently to ADMETlab 3.0 (2024) [86,87,88]. The latest version integrates multi-task directed message-passing neural networks (DMPNNs) to predict 119 ADMET endpoints with enhanced accuracy and robustness, thereby facilitating early-stage screening. With over 3.95 million cumulative uses, the ADMETlab series is now the most widely adopted online platform for drug-likeness prediction [87,89].

In parallel, drug–drug interactions (DDIs) remain a major concern for clinical safety [86], yet existing databases are limited by incomplete coverage, inconsistent evidence hierarchies, and insufficient clinical decision support. To address these limitations, DDInter 2.0 was developed as a comprehensive upgrade to the original DDInter [89,90]. It expands interaction coverage; incorporates drug–food and drug–disease interactions; and, for the first time, integrates therapeutic duplication data. Its enhanced search capabilities and intuitive visualization tools improve the interpretability and applicability of complex interaction profiles, making it a valuable resource for clinicians and researchers that can support safer prescription practices and more informed drug development. The CSM (Cutoff Scanning Matrix) methodology is a computational approach to biological prediction, utilizing the structural and chemical signatures of protein inter-residue distance patterns for predicting feature vectors, enzyme functions [91], and synergistic anticancer drug combinations.

Importantly, AI-driven tools such as ProTox-II also play a pivotal role in predicting the toxicological potential of natural products, which often display a dual nature as both pharmacologically active agents and potential toxicants [92,93]. By facilitating the early identification of compounds with favorable ADMET and toxicity profiles, AI enables the most promising candidates to be prioritized, ultimately enhancing the likelihood of success in downstream stages of drug development [94]. Similarly, several tools have proven to be helpful for predicting bioactivity and synergy between molecules and targets [95,96,97].

### 3.3. Lead Optimization and De Novo Design

Arguably the most innovative and transformative application of artificial intelligence (AI) in preclinical research lies in its capacity to design and optimize molecules de novo. This directly addresses long-standing challenges associated with natural products (NPs), including their limited availability and inherently low yields [98]. Rather than depending on nature to provide optimal bioactive scaffolds, AI-driven methodologies can generate entirely novel chemical entities with predefined characteristics, which can subsequently be optimized to enhance their efficacy, selectivity, and ADMET properties [99]. OptADMET is a web-based platform that can improve the ADMET properties of compounds through substructure mediation, containing around 41,779 validated modifications rules from the 177,191 experimental datasets and additional 146,450 rules from the molecular prescription of 239,194 covering 32 properties around 41,779 validated modification rules from 177,191 experimental datasets and an additional 146,450 rules from the molecular prescriptions of 239,194 datasets, covering 32 properties [100].

This advancement has been facilitated by generative AI frameworks such as Generative Adversarial Networks (GANs) and Variational Autoencoders (VAEs). By learning the latent chemical rules embedded in existing molecular datasets, they can subsequently generate novel, synthetically feasible compounds [101,102,103]. Beyond initial generation, AI supports key optimization strategies, including “group modification,” in which small, targeted structural alterations are introduced, and “scaffold hopping,” where entirely new scaffolds are created while preserving the intended biological activity [104,105].

Reinforcement learning (RL) represents a particularly powerful paradigm within molecular design. In this framework, a generative agent is trained to propose novel chemical structures and is subsequently rewarded or penalized according to how closely the generated molecules satisfy pre-specified pharmacological or physicochemical criteria [106]. This closed-loop, iterative refinement enables the agent to systematically explore chemical space and progressively improve the quality of proposed candidates. Such an approach signifies a paradigm shift in preclinical development, replacing the conventional linear, trial-and-error methodology with an iterative, goal-oriented design cycle [107].

Stokes and colleagues employed a conditional generative adversarial network (GAN) to design the novel antibiotic Halicin, completing the entire workflow from virtual generation to in vitro validation within 48 h, nearly 100-fold faster than conventional approaches [108]. Halicin, structurally distinct from traditional antibiotics, exhibits broad-spectrum activity against multidrug-resistant pathogens, including Mycobacterium tuberculosis and carbapenem-resistant Enterobacteriaceae. It demonstrated therapeutic efficacy in murine models of Clostridioides difficile and extensively drug-resistant Acinetobacter baumannii infections. By training a deep learning model on antibacterial activity prediction, the team screened multiple chemical libraries and identified Halicin from the Drug Repurposing Hub. Further, from 107 million molecules in the ZINC15 database, their framework identified eight structurally novel antibacterial agents, underscoring the potential of deep learning to expand the antibiotic arsenal through the discovery of noncanonical scaffolds [109,110]. This case is seminal because it demonstrated a target-agnostic, phenotype-first AI approach, successfully predicting a novel, synthetic chemotype with a distinct mechanism from a relatively small training set. Although Halicin is not a natural product, this case is highly instructive for NP discovery as it validates the power of AI to identify entirely novel scaffolds against pressing challenges like antibiotic resistance.

In the domain of natural products, quercetin emerges as the phytochemical most frequently associated with AI-based applications [111,112]. As a bioactive flavanol, it possesses strong antioxidant and anti-inflammatory properties, with therapeutic relevance for cancer, AIDS, hypertension, and diabetes [113]. Synergistically with kaempferol, quercetin has also demonstrated antiviral activity against SARS-CoV-2 [114,115,116]. Current research leverages AI to optimize plant-based extraction processes, design novel quercetin analogs, and construct predictive models for evaluating its antioxidant and anticancer activities.

Together, these examples illustrate the progression of deep learning and generative modeling from isolated “point breakthroughs” to systematic strategies for rational drug design. By integrating multimodal datasets, dynamic optimization algorithms, and mechanism-informed modeling, AI is transitioning drug discovery from empirical “trial-and-error” toward rational construction, enabling concurrent optimization of efficacy, safety, and developability [117].

Looking forward into the future with all plausibility, it is expected to have a fully integrated pipeline of virtual design with zero human involvement, with robotic synthesis up to experimental feedback, creating a seamless and automated design-make-test-analyze cycle that maximizes both efficiency and innovation [19,57]. The following table (Table 2) consolidates Artificial intelligence tools and their applications in drug development thereby illustrating the stimulating potential of AI in revitalizing this domain.

### 3.4. AI and NP-Based Drug Delivery Systems

**Figure 3:**
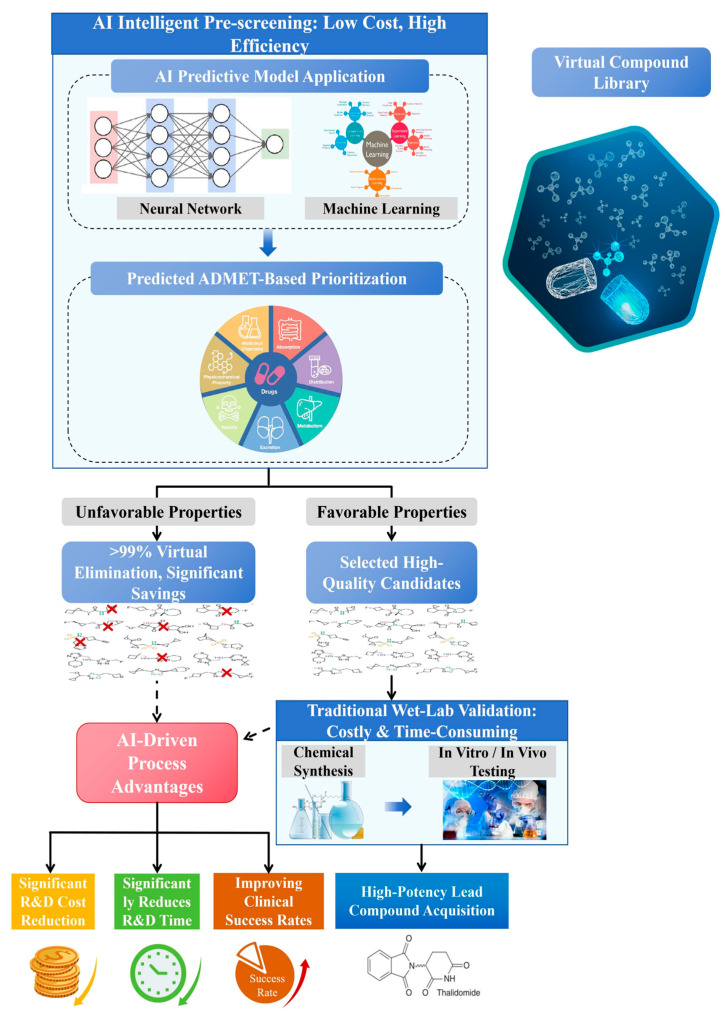
AI-driven in silico ADMET screening: optimizing drug candidate attrition and accelerating development pipeline.

As illustrated in Figure 3, In the field of advanced drug delivery systems, artificial intelligence (AI)-enabled strategies are increasingly employed to refine nanoparticle engineering by systematically optimizing parameters such as particle size, surface functionalization, and release kinetics. These optimizations enhance drug bioavailability while simultaneously reducing off-target effects and systemic toxicity [118]. AI-driven methodologies not only accelerate the drug development pipeline but also support the progression toward precision and personalized medicine. Effective drug delivery remains indispensable for optimizing the pharmacokinetic (PK) and pharmacodynamic (PD) characteristics of therapeutic agents, thereby ensuring improved clinical outcomes. In light of the escalating complexity and cost of new molecular entity (NME) development, the relevance of advanced delivery platforms has expanded significantly [119]. AI technologies now underpin an integrated workflow encompassing the input of disease- and drug-specific data, molecular and physicochemical property-based screening, AI-mediated predictive modeling, and the identification of optimized carrier systems [120,121]. This convergence of computational and experimental approaches holds considerable potential to increase the efficiency, accuracy, and scalability of drug delivery systems (DDSs) design [79].

Traditional liposomal formulations often exhibit inadequate tumor-specific accumulation, constraining their therapeutic efficacy [122]. Recent studies suggest that natural products with dual capabilities of lipid bilayer modulation and tumor-targeting activity can significantly enhance liposomal performance [123]. However, the experimental evaluation of over 300,000 natural product candidates presents formidable challenges in terms of time and resources. Addressing this limitation, researchers from Fudan University’s School of Pharmacy and Shanghai Jiao Tong University established a bidirectional deep learning-integrated platform coupled with experimental validation. This platform successfully identified compounds capable of lipid membrane regulation and glucose transporter 1 (Glut1)-targeting, leading to the development of bifunctional liposomal systems. These advanced nanocarriers demonstrated improved tumor selectivity and therapeutic efficacy in murine models, establishing a paradigm for intelligent DDS engineering via AI-guided prediction and FDA [78].

While many natural products possess potent bioactivity, their translation into therapies can be hindered by poor pharmacokinetics, off-target effects, or inability to reach intracellular sites of action. Adeno-associated virus (AAV) vectors, particularly with engineered capsids, represent a promising delivery platform for NP-derived modalities. For instance, AAV has been used to deliver genes encoding engineered nanobodies that mimic the activity of plant-derived cytotoxins [124], or to express biosynthetic enzymes for the local production of therapeutic terpenoids in animal models [125]. These approaches directly address the delivery challenges of classical NP small molecules. Parallel advances in AAV capsid engineering can further enhance the precision of such strategies. Companies like Dyno Therapeutics employ AI to design capsids with improved tissue specificity and delivery efficiency [126], a methodology that involves generating diverse capsid libraries and using algorithms to predict optimized sequences based on functional maps [127]. Together, the integration of NP-inspired therapeutic cargo with these advanced delivery vectors highlights a promising direction for advancing NP-based therapies.

Fei Li and colleagues employed AI-driven strategies combining combinatorial hydrogel libraries with machine learning to design self-assembling peptide hydrogels exhibiting tunable mechanical properties such as stiffness and elasticity. These hydrogels effectively encapsulate a broad spectrum of therapeutics, including nucleic acids and small molecules, expanding their translational potential [128]. In a related effort, Safa and Samar Damiati utilized artificial neural networks (ANNs) to optimize the drug-loading efficiency of poly (lactic-co-glycolic acid) (PLGA) nanoparticles. The optimized system produced monodispersed PLGA particles encapsulating indomethacin (IND) with controlled morphology, high encapsulation efficiency, and sustained release profiles achieving up to 80% release, underscoring the role of AI in rational nanoparticle design [129].

AI-driven innovation is increasingly embedded across the DDS research pipeline. Pharmaceutical companies such as Medicilon have developed dedicated AI-enabled platforms, including AiLNP, AiRNA, and AiTEM, that streamline nucleic acid screening, DDS optimization, and lipid nanoparticle formulation. These platforms substantially reduce developmental timelines and associated costs, thereby expediting clinical translation [107]. Furthermore, in silico medicine has leveraged graph neural networks (GNNs) to design PD-L1-targeting nanobodies with enhanced receptor-binding affinity, achieving improved tumor-specific accumulation [130]. Complementarily, ETH Zurich has developed an AI-assisted navigation system that integrates real-time ultrasonic imaging to precisely guide magnetic microspheres toward pancreatic tumors. This approach overcomes physiological barriers and enables highly localized therapeutic delivery, representing a promising advance in AI-orchestrated nanomedicine [131].

The integration of nanobodies—single-domain antibody fragments—into natural product (NP) research addresses two fundamental bottlenecks in the field: mechanism elucidation and translational delivery. Their unique biophysical properties, including their small size, high stability, and deep tissue penetration, make them uniquely suited for this role [132].

Nanobodies provide a crucial bridge between the bioactivity of natural products (NPs) and modern biologic therapeutics, a strategy exemplified by the development of NP-derived nanomedicines. Their application follows two principal pathways: First, they can serve as highly specific targeting moieties to deliver potent, cytotoxic NP-derived payloads (e.g., maytansinoid conjugates) directly to diseased cells. This approach, central to several antibody–drug conjugates (ADCs), minimizes systemic toxicity by enhancing tumor-specific accumulation [133]. Second, their hypervariable loops can be engineered to mimic the essential pharmacophore of an NP, creating a stable, protein-based equivalent. This “biologization” strategy transforms NPs with inherently poor drug-like properties into viable therapeutic candidates with superior pharmacokinetics [134,135]. The integration of NP-inspired pharmacophores with the targeting prowess of nanobodies aligns with and advances the emerging paradigm of targeted NP–biologic conjugates for next-generation nanomedicines.

## 4. AI in Clinical Translation and Post-Market Surveillance for NPs

### 4.1. Drug Repurposing of NPs

The exorbitant cost and extended timelines associated with de novo drug discovery have positioned drug repurposing as a transformative strategy in contemporary biopharmaceutical research. This approach, which seeks to identify novel therapeutic indications for existing pharmacological agents, markedly reduces development time and financial risk by capitalizing on established safety and pharmacokinetic data [136]. Artificial intelligence (AI) has revolutionized this traditionally serendipitous practice, converting it into a systematic, highly efficient, and data-driven methodology [135].

AI achieves this by employing advanced machine learning and deep learning models capable of integrating and analyzing vast, heterogeneous datasets encompassing genomic, proteomic, pharmacological, and clinical information [66]. These algorithms can discern subtle patterns and latent associations between chemical entities and biological targets that remain imperceptible to human analysis [137]. For instance, natural language processing (NLP) methods can systematically mine the scientific literature and patent databases, extracting semantic relationships that reveal previously unrecognized drug–disease connections [138]. Through such data-driven strategies, AI enables the rapid identification of repurposing candidates at a scope and scale unattainable by conventional approaches [139].

Natural products (NPs) represent particularly compelling candidates for repurposing owing to their long-standing safety records derived from traditional use, alongside their inherent pleiotropic and multi-target pharmacology, features especially advantageous for addressing complex, multifactorial pathologies [140,141].

The bioactivity of many NPs is critically dependent on their absolute stereochemical configuration. However, commonly used 2D molecular fingerprints fail to distinguish between different stereoisomers, potentially leading to the oversight of active compounds. Furthermore, NPs, especially macrocycles, often exhibit significant conformational flexibility. Traditional molecular docking methods may fail to accurately identify the optimal bioactive conformation for target binding, resulting in erroneously low scores and false negatives [64].

NPs are characterized by highly complex and diverse three-dimensional scaffolds. Most molecular descriptors were developed for structurally “flatter” synthetic drug-like molecules and are often inadequate for accurately capturing the unique structural features of NPs [142]. This underscores the need to develop NP-tailored molecular representations or to adopt models like Graph Neural Networks, which can learn directly from molecular graphs.

Predominant VS compound libraries are primarily composed of synthetic molecules. Evaluating NPs within this context creates an unfair comparison and biases the screening process toward familiar chemical archetypes. Effective NP-focused VS requires the use of specialized, NP-centric databases [132]. However, these databases are typically smaller in scale and sparser in bioactivity annotations, presenting a significant bottleneck for training robust AI models. Transfer learning—pre-training models on large-scale synthetic data followed by fine-tuning with limited NP data—is currently an effective strategy to mitigate this data scarcity.

AI’s role extends beyond therapeutic compound optimization to deciphering the fundamental biosynthesis of complex NPs. A prime example is the study of saxitoxin, a potent marine neurotoxin. Researchers employed AI-driven comparative genomics to predict its biosynthetic gene clusters across dinoflagellate species and model the evolutionary trajectory of its pathway [143]. This case highlights AI’s power in transforming genomic data into testable hypotheses about NP origin and diversification, forming a knowledge foundation for future bioengineering and discovery. A multi-step AI workflow combining network pharmacology, deep learning-based docking, and molecular dynamics simulations identified active anti-fibrotic flavonoids from a Traditional Chinese Medicine formula, demonstrating its efficacy through a mechanistic framework [144]. This represents a systems pharmacology approach which links complex mixtures to molecular targets.

Notably, AI-based approaches have been employed to predict novel therapeutic applications for compounds such as quercetin, a plant-derived flavanol with well-documented antioxidant and anti-inflammatory properties, in the treatment of conditions such as COVID-19 and cancer. Moreover, AI can forecast potential synergistic interactions between natural compounds, thereby informing the rational design of combination therapies [90,145,146,147]. These cases confirm that AI is a versatile tool, but underscore that success depends on high-quality training data and efficient experimental validation. They illustrate different strategic applications—phenotypic screening, Lead Optimization, and systems pharmacology—which provide a roadmap for integrating AI into specific stages of NP-based drug discovery. Collectively, these advancements underscore the untapped potential of NPs as a fertile resource for AI-driven drug repurposing initiatives.

### 4.2. Personalized Phytotherapy and Precision Medicine

The overarching objective of artificial intelligence (AI) in medicine is to facilitate a paradigm shift from conventional “one-size-fits-all” therapeutic strategies toward individualized treatment approaches. Such a framework, commonly referred to as personalized or precision medicine, seeks to deliver more effective interventions with reduced adverse effects by accounting for a patient’s unique genetic background, clinical history, and lifestyle factors [148]. Within the domain of natural products (NPs), AI is catalyzing the emergence of a new paradigm of “personalized phytotherapy” [20,149].

AI achieves this by integrating and analyzing heterogeneous patient-derived datasets, including electronic health records (EHRs), genomic profiles, wearable sensor outputs, and multi-omics information [150,151]. Through advanced computational modeling, AI algorithms can delineate individual molecular signatures and forecast patient-specific therapeutic responses, thereby enabling optimization of dosage regimens and the development of targeted treatment strategies [78,152].

An illustrative application can be found in Traditional Chinese Medicine (TCM), which has long emphasized a multiparametric evaluation of patient conditions [149,153]. Contemporary AI systems are being deployed to enhance this process by systematically analyzing symptomatology, medical history, and biometric parameters to recommend individualized herbal formulations and acupoint prescriptions, thus aligning ancient therapeutic principles with modern data-driven precision frameworks. Moreover, the advent of decentralized AI methodologies, such as federated learning, is addressing critical challenges related to data privacy and security [154,155]. These approaches allow predictive models to be trained across large-scale, distributed datasets without necessitating the exchange of raw patient information, thereby safeguarding confidentiality while maintaining analytical robustness.

### 4.3. Quality Control and Standardization

Maintaining the purity, potency, and safety of natural products (NPs) remains a formidable challenge due to their intrinsic variability and vulnerability to adulteration [156]. Artificial intelligence (AI) has emerged as a pivotal tool in overcoming these limitations, enabling an unprecedented degree of precision in quality assurance and standardization.

AI augments conventional analytical methodologies, including spectroscopy and chromatography, by deploying machine learning algorithms to interrogate the extensive datasets they generate [157,158]. These algorithms are capable of extracting distichemicali “fingerprintsi”, thereby facilitating accurate discrimination between authentic and adulterated herbal materials [142]. For example, the integration of hyperspectral imaging with machine learning classifiers has demonstrated over 98% accuracy in detecting adulteration in commodities such as honey [159]. In addition to chemical profiling, deep learning models trained on large-scale plant image repositories can visually authenticate botanical species, effectively distinguishing even closely related taxa with high precision and efficiency [160,161].

**Figure 4:**
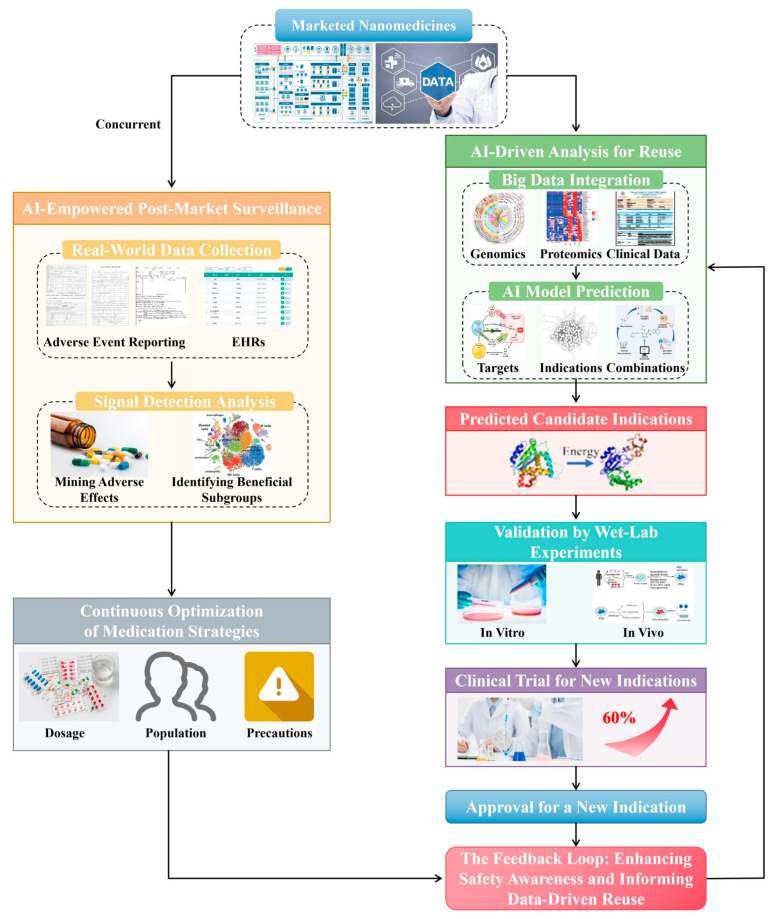
AI-driven lifecycle management of marketed nanomedicines: from post-market surveillance to data-informed repurposing and optimization.

Beyond laboratory-based analyses, AI is being coupled with blockchain technology to establish secure, transparent, and tamper-proof supply chains for NPs. By generating an immutable digital ledger that traces a product from its point of origin to end-user delivery, AI-enabled verification at multiple checkpoints mitigates counterfeiting, ensures compliance with ethical sourcing practices, and preserves product integrity across the entire distribution network [162]. As shown in Figure 4, This integrated ecosystem, which interconnects plant sourcing with patient administration, leverages multiple layers of AI applications that collectively reinforce transparency, safety, and therapeutic efficacy. It is critically important to mention that the ability to track the complete trajectory of a natural product, from harvest through patient delivery, is essential for advancing personalized phytotherapy, as it guarantees that individuals receive standardized, high-quality preparations tailored to their specific therapeutic requirements [163].

## 5. Challenges and Enabling Infrastructure

### 5.1. Data Ecosystem

The success of any AI system relies on the quality and availability of data, as is the case for its usage in NP drug discovery. At present, the data landscape on NPs constitutes a major bottleneck to progress, as available resources remain highly fragmented, inconsistently curated, and dispersed across multiple, frequently siloed repositories [164]. These datasets are inherently multimodal, encompassing genomic sequences, metabolomic signatures, spectral readouts, and unstructured textual records, yet they lack standardized formats and harmonized ontologies [165]. The absence of standardization and interoperability severely restricts compatibility with contemporary deep learning architectures, which are optimized for processing clean, structured, and uniform data inputs [166,167].

Compounding this challenge is the scarcity of high-quality, annotated datasets specific to NPs, making effective model training more difficult [168]. This deficiency contributes to algorithmic pitfalls such as overfitting, whereby models demonstrate high performance on training data yet fail to generalize to novel or unseen inputs [169]. The resulting data fragmentation hinders the ability of AI frameworks to extract cross-modal patterns that connect structural, functional, and pharmacological dimensions, thereby constraining their ability to predict novel chemotypes or uncover emergent bioactivities [170]. Addressing this limitation will necessitate the development of a unified, interoperable repository capable of systematically linking and cross-referencing all modalities of NP-related data, from molecular structures and bioactivity profiles to ethnopharmacological knowledge [171] in order to unlock the full potential of AI-driven NP drug discovery.

### 5.2. Algorithmic and Methodological Hurdles

The obstacles facing artificial intelligence (AI) usage in natural product (NP) research extend beyond data-related limitations to encompass substantial algorithmic and methodological challenges [172]. A particularly critical issue is the “black box” nature of advanced deep learning models, wherein their internal decision-making processes remain opaque and lack interpretability [173]. This opacity is a significant barrier in a field that demands rigorous, transparent, and verifiable evidence to meet the stringent requirements of regulatory approval and clinical translation. In the absence of mechanistic interpretability, researchers are unable to fully trust model-generated predictions.

The practical hurdles of each paradigm are significant. Ligand-based approaches, while efficient, are notoriously prone to “scaffold bias”, often memorizing training set chemotypes rather than learning generalizable rules, leading to poor performance on novel NP scaffolds [174]. Structure-based methods like docking struggle with the conformational flexibility of many NPs and the inaccuracy of scoring functions for non-drug-like molecules, frequently resulting in false negatives for genuine binders. Although deep learning models (e.g., Graph Neural Networks) promise to overcome these issues, their “black box” nature and extreme sensitivity to hyperparameters and data splits raise major reproducibility concerns, making their predictions difficult to trust and validate prospectively. The field of de novo generation grapples with the synthetic intractability of its outputs; a landmark study found that a substantial fraction of AI-designed molecules were deemed unrealizable by expert chemists, highlighting a critical disconnect between computational optimization and practical synthesis. Even the promising paradigm of multi-omics integration is bottlenecked by the scarcity of large, paired, and standardized datasets (e.g., linking genomic clusters directly to isolated metabolites and their bioactivity), which are essential for training robust models [174].

As summarized in Table 3, different AI methodologies in drug discovery exhibit distinct key strengths and major limitations, which are comparatively analyzed in detail. For instance, virtual screening enables rapid analog or novel scaffold discovery but suffers from scaffold bias or scoring inaccuracies; de novo generation explores novel chemical space yet faces challenges in synthetic tractability; ADMET prediction supports early attrition risk assessment but is constrained by data quality; explainable AI enhances transparency while risking non-unique explanations; and integrated systems model complex biology but require heterogeneous data for validation.

Therefore, a critical appraisal reveals that no single AI methodology is a universal solution. The choice must be strategic, dictated by the specific research question, data availability, and the stage of the discovery pipeline. Acknowledging and rigorously testing against these methodological bottlenecks—through practices like scaffold-split validation, prospective experimental confirmation, and adherence to application domain boundaries—is paramount for advancing robust, reproducible, and impactful AI-driven NP research.

Beyond individual tools, the field faces systemic challenges. Comparative methodological weaknesses are evident: ligand-based models suffer from scaffold bias and depend heavily on training set quality [14,179,180], while structure-based methods struggle with the flexibility of NPs. The foundational data itself is problematic; public bioactivity databases underrepresent NP chemotypes, while NP-specific resources often contain noisy or non-standardized data [175,176,177], creating a “garbage in, garbage out” risk. Furthermore, many studies only report optimistic internal validation metrics, neglecting the critical need for prospective external testing and clear definition of the model’s applicability domain—the chemical space where its predictions are reliable [178]. This over-reliance on convenient but flawed benchmarks, combined with a frequent disconnect between computational hit identification and practical experimental validation, forms a major translational gap that must be bridged for the field to mature.

In addition, contemporary AI systems face intrinsic constraints in their capacity to extrapolate beyond known chemical and enzymatic landscapes [181]. While these models are highly effective at identifying patterns and relationships within established chemical space, their ability to predict genuinely novel chemistries or previously uncharacterized enzyme functions remains limited [182]. This limitation underscores the necessity of adopting human-in-the-loop strategies and hybrid frameworks that integrate AI-driven computational capabilities with the domain expertise, creativity, and critical reasoning of researchers. Such synergistic approaches are essential to overcome the boundaries of current methodologies and to advance AI-driven discovery in NP research.

### 5.3. Critical Data Hurdles: Bias, Noise, and the Path to FAIR Data

The reliability of AI models in NP discovery is fundamentally constrained by the “garbage in, garbage out” principle, with data quality being a primary bottleneck. Systematic biases in public repositories (e.g., ChEMBL), which are dominated by synthetic compounds and single-target assays, lead to a severe underrepresentation of NP chemical space and polypharmacology. Furthermore, heterogeneous data from diverse sources—such as crude extracts, varied bioassays, and traditional records—suffer from a lack of standardized annotation, introducing noise and making machine learning integration profoundly challenging [183].

To build robust models, researchers in the field must prioritize data-centric solutions. Adopting the FAIR principles is essential. Community-driven resources like the “MIBiG database” for biosynthetic gene clusters demonstrate the value of enforced curation standards. Meticulous “preprocessing of raw data” is also critical; for example, optimized preprocessing reduced false positive rates in a cannabis provenance study from 21 to 27% to 11–14%. Ultimately, advancing AI in NP discovery requires a paradigm shift where investment in high-quality, standardized, and ethically curated data is recognized as the indispensable foundation of all computational progress [184].

### 5.4. Ethical, Regulatory, and Sustainability Considerations

**Figure 5:**
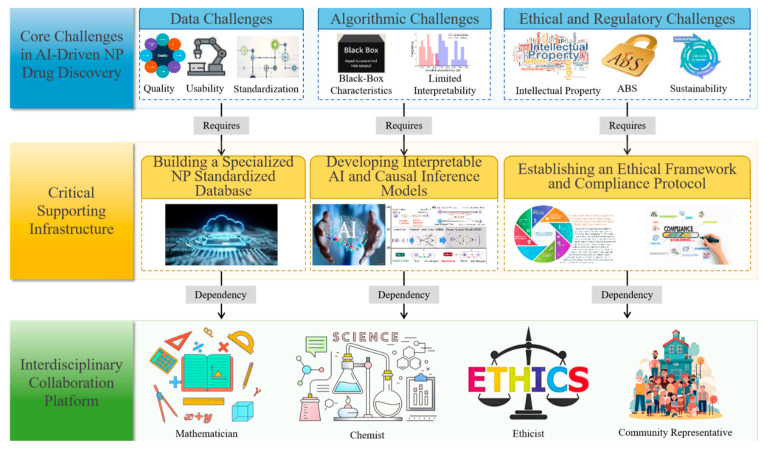
Addressing core challenges in AI-driven natural product drug discovery: from critical infrastructure to interdisciplinary collaboration.

As depicted in Figure 5, the integration of artificial intelligence (AI) with traditional knowledge and natural resources introduces a distinct set of ethical, regulatory, and sustainability challenges that diverge considerably from those encountered in conventional drug discovery. A central issue concerns the intellectual property rights of Indigenous communities, whose knowledge systems have been transmitted across generations [33]. The application of AI to mine such knowledge in the absence of a robust ethical framework risks both the exploitation and “digital marginalization” of traditional practitioners [33]. To mitigate this, it is imperative that AI-driven research be designed to empower these communities through inclusive practices and equitable benefit-sharing mechanisms.

A cornerstone of this discussion is the Nagoya Protocol on Access and Benefit-Sharing (ABS). While it establishes a vital legal framework for Prior Informed Consent (PIC) and Mutually Agreed Terms (MATs), its practical implementation reveals significant complexities. A salient case study involves the alkaloids from *Mitragyna speciosa* (kratom), a plant with a long history of traditional use in Southeast Asia. The rapid global commercialization of kratom-derived products has largely occurred outside any formal ABS framework, triggering international regulatory disputes and raising critical questions about benefit and risk distribution in the commodification of traditionally managed species [185]. This case underscores the gap between international agreements and on-the-ground governance.

Robinson extends the debate to the digital realm, arguing that using digitized TK in databases—a potential feedstock for AI—creates new obligations, necessitating “digital PIC” and traceability mechanisms absent in most platforms [186]. This analysis reveals a core tension: profound knowledge asymmetry. AI models can efficiently mine bioactivity patterns from TK-associated compounds, yet the current data ecosystem is ill-equipped to recognize the value of knowledge holders or ensure justice for them, risking a form of digital bioprospecting.

Another critical consideration is the preservation of “living knowledge” held by herbalists and traditional healers. AI systems, which are typically limited to codified and structured data, may overlook subtle contextual factors and experiential safety insights embedded within traditional practices. Over-reliance on algorithmic outputs without adequate understanding of the original knowledge base could result in serious errors, such as neglecting warnings regarding the toxicity of botanicals when improperly prepared [187]. Thus, the integration of AI must complement practitioner expertise rather than replace it.

It is equally important to ensure that AI serves as an instrument for advancing environmental sustainability, rather than undermining it. AI technologies can be leveraged to address ecological challenges, including overharvesting and habitat loss, by analyzing genomic and ecological datasets to identify alternative, sustainable sources of natural products. Furthermore, AI-driven agricultural innovations, such as precision cultivation and controlled-environment farming, enable growth conditions to be optimized while minimizing land use, pesticide application, and water consumption [188]. Collectively, these strategies can ensure that the resurgence of NP-based drug discovery is not only technologically sophisticated but also ethically responsible and environmentally sustainable [189].

## 6. Conclusions and Future Course

### 6.1. Critical Appraisal: Limitations, Failures, and Unresolved Challenges

A fundamental challenge in this field stems from biases embedded within widely used bioactivity datasets, such as ChEMBL, which are dominated by synthetic compounds and high-throughput screening (HTS) data. This can lead to “analog bias,” where models learn to associate simple chemical fingerprints with activity labels rather than genuine structure–activity relationships. Consequently, they may over-predict the activity of molecules that have similar fingerprints to known actives in the training set, generating false positives. A systematic evaluation demonstrated that models achieving excellent performance in random cross-validation often see a dramatic drop in accuracy when assessed via more realistic temporal or scaffold splits, designed to simulate prospective prediction of novel chemotypes [181]. For NP discovery, where chemical space differs significantly from synthetic libraries, this bias is particularly acute. Models validated only by internal (random) metrics may provide a false sense of security, and their predictions for novel NP scaffolds require rigorous external validation.

The ultimate test for an AI model in NP discovery is its performance on truly novel, structurally distinct natural scaffolds—precisely the “out-of-distribution” (OOD) data where many models falter. A study on antimicrobial activity prediction illustrated this gap: a model performed well on standard benchmark sets but exhibited significantly degraded performance (e.g., a drop in AUC-ROC) when applied to an independent test set of marine-derived NPs featuring complex macrocyclic and polyketide architectures [190]. This failure highlights a core paradox: the most therapeutically interesting NPs are often those farthest from the training data distribution. Many contemporary AI models excel at interpolation within known chemical space but struggle with extrapolation to the unique structural motifs characteristic of many NPs. This underscores the non-negotiable requirement for external validation on diverse, NP-centric compound sets as a minimum standard for assessing translational utility.

While generative models (e.g., GANs and VAEs) can optimize molecules for computationally driven objectives like predicted binding affinity or quantitative estimate of drug-likeness (QED), the generated structures often lack synthetic tractability. A landmark study found that a substantial fraction of AI-generated molecules were rated as difficult or impossible to synthesize by expert medicinal chemists [191]. For NP-like molecules, the challenge is compounded by complex stereochemistry and intricate ring systems. Optimizing for simplistic scores without embedding hard constraints from retrosynthetic analysis can yield molecules that are computationally elegant but practically unrealizable. This disconnect necessitates the tighter integration of generative AI with synthesis-aware algorithms, prioritizing synthetic feasibility and stereochemical soundness from the earliest design stages.

Many NPs exert their therapeutic effects through polypharmacology—modulating multiple targets within a biological network. However, most AI models are designed for single-target activity prediction and are ill-equipped to capture these synergistic, system-level effects. Attempts to predict multi-target profiles or downstream phenotypic outcomes often result in low accuracy and misleading associations [192]. This is a significant limitation of the prevailing reductionist AI approach when applied to NPs, whose value may lie in their network pharmacology. Predicting the nuanced, often beneficial side-effect profiles of NPs remains a formidable challenge, highlighting the need for novel AI paradigms that incorporate systems biology and phenotypic screening data.

### 6.2. Towards Rigorous Science: Reproducibility and Benchmarking

#### 6.2.1. The Reproducibility Challenge

For AI to transition from a promising research tool to a trusted component of NP discovery pipelines, resolving its reproducibility crisis and establishing rigorous, standardized benchmarking are critical. Addressing these issues is fundamental to building a reliable and cumulative knowledge base.

The replication of published studies on AI-powered NP discovery is frequently impeded by several interconnected factors. A primary obstacle is the lack of standardized benchmarks. Many studies utilize proprietary, non-public, or inconsistently curated datasets, rendering direct comparison impossible. This problem is exacerbated by the prevalent non-disclosure of critical materials, including source code, exact compound structures, and the specific data splits used for model training and validation. Without access to these, independent verification is unfeasible. Furthermore, the performance of complex AI models is highly sensitive to hyperparameter configurations and the random initialization used for data partitioning—details that are often insufficiently reported. An insightful analysis demonstrated that the superior performance reported for novel models could often be replicated or surpassed by standard baselines through meticulous hyperparameter optimization alone, underscoring how incomplete reporting can distort perceived progress in the field [193]. In NP research, where data is inherently sparse and heterogeneous, these issues are magnified. The reported success of a model may be an artifact of a favorable data split that included chemically similar training and test compounds, rather than a true indicator of its ability to generalize to novel, structurally unique NP scaffolds. Collectively, these practices hinder the fair comparison and robust advancement of methodologies.

#### 6.2.2. The Imperative for Comparative Benchmarking

To substantiate claims of added value, AI approaches must be evaluated using head-to-head comparisons against established traditional discovery methods under stringent, transparent conditions. Assertions of “accelerated discovery” or “improved success rates” remain ambiguous without a definitive baseline. It is important to contextualize these reported accelerations; they often pertain to the in silico phase and do not account for downstream experimental timelines, which remain a major bottleneck.

Valid benchmarking requires comparison against established pillars of NP discovery, such as bioactivity-guided fractionation of crude extracts, high-throughput screening (HTS) of physical compound libraries, and structure-based virtual screening using molecular docking [175].

Evaluations must employ consistent, blinded metrics on shared, publicly accessible datasets. The field must progress beyond reliance on internal validation metrics and adopt prospective, temporal, or scaffold-split validation protocols that simulate real-world discovery scenarios targeting novel chemical entities. These improved metrics, while encouraging, are often achieved under optimized conditions on benchmark datasets. Their generalization across diverse target classes and chemical spaces requires further extensive validation.

Encouragingly, community-driven initiatives are emerging to set these standards. While general-purpose resources like the MoleculeNet benchmark suite provide a foundation for open comparisons when predicting molecular properties [2], the development of NP-specific benchmarking challenges is crucial. The establishment of curated, blinded NP datasets for community-wide algorithm testing would represent a significant step forward. The adoption of such frameworks, coupled with strong data and code sharing mandates from journals and funders, is an essential prerequisite for the field’s maturation.

#### 6.2.3. Synthesis and Path Forward

Confronting reproducibility and benchmarking issues is central to demonstrating the scientific credibility of AI usage in NP discovery. Embracing a culture of open science and rigorous validation is paramount; only through such concerted efforts can the field evolve from publishing isolated demonstrations of potential to generating robust, reproducible, and comparative evidence. This will clearly delineate when and how AI can provide a definitive advantage over conventional methodologies in unlocking the therapeutic potential of natural products [194].

The integration of artificial intelligence (AI) has initiated a profound paradigm shift, reinvigorating the domain of natural product (NP) drug discovery and enabling researchers to surmount longstanding barriers that have historically impeded its progress [19]. As outlined throughout this review, AI provides advanced computational solutions that can accelerate every stage of the drug development continuum, from the initial identification of bioactive leads to clinical translation and post-market surveillance. By facilitating systematic, data-driven exploration of an expansive and chemically diverse molecular space, AI unlocks the vast therapeutic potential embedded within nature’s chemical repertoire. This transformative capacity not only reduces temporal and financial burdens but also heralds a new era of drug development that is more efficient, precise, and patient-centered.

First, evolution toward an integration of multi-omics systems with AI is essential. In order to move beyond models that are solely reliant on chemical structure, future systems must be able to perform joint learning using genomic (biosynthetic potential), metabolomic (compound profiles), and phenotypic data streams in order to enable true end-to-end discovery pipelines. Second, adopting collaborative frameworks such as Federated Learning can overcome data silos by enabling model training across decentralized, proprietary datasets without sharing raw data, thus fostering pre-competitive collaboration while preserving intellectual property [195]. Third, establishing community-driven, NP-specific open-source benchmarks is fundamental for ensuring reproducibility and rigor, shifting the focus from algorithmic novelty to solving robust, real-world problems. Finally, ethical principles must be translated into practice through actionable technical tools, such as digital provenance tracking for traditional knowledge and frameworks for equitable benefit sharing, developed in collaboration with legal and Indigenous data sovereignty experts [196].

Ultimately, the greatest impact will stem from the strategic convergence of these pillars: building powerful, privacy-aware AI models trained on federated multi-omics data, rigorously validated against open benchmarks, and inherently designed to guide equitable and transparent discovery. By steering efforts toward these integrative, collaborative, rigorous, and ethically embedded pathways, the research community can ensure AI’s role as a powerful engine for discovering the next generation of sustainable and equitable natural product-inspired therapeutics.

### 6.3. Future Perspectives on AI-Driven Natural Product Discovery

In the future, AI-driven NP discovery is poised to show even greater advancements, catalyzed by innovations in computational modeling and interdisciplinary collaboration. Foundational models such as AlphaFold, which has revolutionized protein structure prediction, will serve as springboards for more specialized applications in chemistry and biology, democratizing access to cutting-edge tools and markedly reducing research costs [52]. In future developments, the establishment of fully integrated, closed-loop systems encompassing virtual molecular design, robotic synthesis, and automated experimental feedback is critical [41]. Guided by reinforcement learning, such workflows will enable the continuous, autonomous optimization of candidate compounds, thereby accelerating the drug discovery cycle to an unprecedented pace. Fully autonomous discovery remains a long-term goal, as current systems are best described as powerful decision-support tools that augment, rather than replace, expert intuition and experimental validation.

The convergence of AI with complementary emerging technologies, such as organ-on-chip platforms and quantum computing, further augments this vision [197,198,199,200]. These synergies promise enhanced accuracy in predicting drug behavior and more comprehensive modeling of biological complexity, significantly reducing false positives within the development pipeline [199]. To achieve widespread adoption and regulatory acceptance, however, it is imperative to address issues with the interpretability of current deep learning systems. Developing explainable AI (XAI) frameworks will provide insight into algorithmic decision-making, thereby fostering scientific trust and facilitating integration into stringent regulatory processes [201]. Equally important is the establishment of robust ethical frameworks that balance technological innovation with the protection of traditional knowledge systems, cultural heritage, and ecological sustainability [197].

## Figures and Tables

**Table 1:** Artificial intelligence-driven solutions to traditional bottlenecks in natural product drug discovery: key technologies and references.

Traditional Bottleneck	AI-Driven Solution	Key AI Technology	Ref.
Time-consuming isolation and characterization	Spectral data analysis, automated workflow	Deep Neural Networks and Computer Vision	[19]
Dereplication, redundant discovery	AI-powered databases, classification and clustering	Unsupervised Learning (e.g., K-means)	[19]
Poor Drugability (solubility and bioavailability)	In silico AMET prediction	Graph Neural Networks, QSAR models	[22]
Limited supply and low yields from source	Biosynthetic engineering via in silico design	Reinforcement Learning, Generative Models, Variational Autoencoders	[23]
Inadequate understanding of mechanisms	Multi-Omics data integration and Network analysis	Deep (Reinforcement) Learning and Knowledge Graphs	[24]

**Table 2:** Artificial intelligence tools and their applications in the preclinical drug development pipeline: algorithms, functions, and references.

Stage of Preclinical Pipeline	AI Tool/Model	Underlying Algorithm(s)	Function/Application	Applicability Domain and Notes	Validation Level	Typical Data Requirements	Ref.
Target Prediction	SPiDER, STarFish, TiGER	Self-organizing maps, Ensemble methods (RF, k-NN)	Identifies innovative molecules and their targets; predicts drug side effects and repurposing options	General small molecules; not specific to natural products.	Primarily in silico; some tools have limited experimental validation.	Chemical structures, bioactivity databases, and omics data.	[68,69,70]
ADMET Screening	ADMET-AI, PiscesCSM, ProTox-II	Graph Neural Networks, Machine learning models	Predicts absorption, distribution, metabolism, excretion, and toxicity; filters vast chemical libraries for druggability	General chemical libraries; applicability may vary with chemical space.	Mostly in silico; some tools benchmarked with experimental datasets.	Molecular descriptors, SMILES strings, and historical ADMET data.	[81,85,86,87,88,90]
Bioactivity and Synergy Prediction	CCSynergy, SynAI, SynPred	Deep Neural Networks (DNNs), QSAR	Predicts and ranks the biological activities of compounds; predicts synergistic interactions for combination therapies	General drug pairs; limited validation for natural product combinations.	Predominantly in silico; few tools validated in cell-based assays.	Dose–response matrices, drug structures, genomic profiles.	[95,96,97,98]
Lead Optimization and De Novo Design	GANs, VAEs, OptADMET	Generative AI, Reinforcement Learning	Designs novel molecules with optimized properties; refines molecular synthesis paths through iterative learning	General de novo design; may require tuning for natural product-like chemical space.	Proof-of-concept in silico; experimental validation rare.	Chemical libraries, property labels (e.g., solubility, potency).	[95,96,114]

**Table 3:** Comparative analysis of AI methodologies in drug discovery.

Methodology	Key Strengths	Major Limitations	Ref.
Virtual Screening	LBVS: Fast, efficient for analog discovery. SBVS: Target-agnostic, enables novel scaffold discovery.	LBVS: High scaffold bias, poor generalization. SBVS: Challenged by flexibility/scoring inaccuracies.	[14]
De Novo Generation	Explores novel chemical space; enables multi-property optimization.	Outputs often lack synthetic tractability; validation is complex.	[175]
ADMET Prediction	Enables early attrition risk assessment; cost-efficient.	Models limited by data quality/coverage; unreliable for novel chemotypes.	[176]
Explainable AI (XAI)	Increases trust and transparency; provides actionable insights for chemists.	Explanations can be non-unique; may reduce model performance.	[177]
Integrated Systems	Models complex biology; links molecular to phenotypic effects.	Requires heterogeneous data; complex to build and validate.	[178]

## Data Availability

The original contributions presented in this study are included in the article. Further inquiries can be directed to the corresponding author.
